# The contributions of relative brain viscosity to brain function and health

**DOI:** 10.1093/braincomms/fcae424

**Published:** 2024-12-03

**Authors:** Grace McIlvain

**Affiliations:** Department of Biomedical Engineering, College of Engineering and Applied Sciences, Columbia University, New York, NY 10027, USA; Department of Radiology, Vagelos College of Physicians and Surgeons, Columbia University, New York, NY 10032, USA

**Keywords:** viscosity, elastography, damping ratio, phase angle, spring-pot

## Abstract

Magnetic resonance elastography has emerged over the last two decades as a non-invasive method for quantitatively measuring the mechanical properties of the brain. Since the inception of the technology, brain stiffness has been the primary metric used to describe brain microstructural mechanics. However, more recently, a secondary measure has emerged as both theoretical and experimental significance, which is the ratio of tissue viscosity relative to tissue elasticity. This viscous-to-elastic ratio describes different but complementary aspects of brain microstructural health and is theorized to relate to microstructural organization, as opposed to stiffness, which is related to tissue composition. The relative viscosity of brain tissue changes regionally during maturation, aging and neurodegenerative disease. It also exhibits unique characteristics in brain tumours and hydrocephalus, and is of interest for characterizing traumatic head impacts. Most notably, regional measures of relative brain tissue viscosity appear to hold a unique role in describing cognitive function. For instance, in young adults, relatively lower hippocampal viscosity compared to elasticity repeatedly and sensitively relates to spatial, declarative and verbal memory performance. Importantly, these same trends are not found with hippocampal stiffness, or hippocampal volume, highlighting a potential sensitivity of relative viscosity to underlying cellularity that contributions to normal healthy brain function. Likewise in young adults, in the orbitofrontal cortex, lower relative viscosity relates to better performance on fluid intelligence tasks, and in the Broca’s area of children ages 5–7, lower relative viscosity is indicative of better language performance. In these instances, this ratio shows heightened sensitivity over other structural MRI metrics, and importantly, provides a quantitative and intrinsic alternative to measuring structure–function relationships with task-based fMRI. There are ongoing efforts to improve the accuracy and repeatability of the relative viscosity measurement, and much work is needed to reveal the cellular underpinning of changes to tissue viscosity. But it appears clear that regionally measuring the viscous-to-elastic ratio holds the potential to noninvasively reveal an aspect of tissue microstructure that is clinically, cognitively and functionally relevant to our understanding of brain function and health.

## Teaser

Magnetic resonance elastography is a non-invasive method for measuring brain mechanical properties—traditionally stiffness. Recently however, the ratio of brain viscosity-to-elasticity, (also measurable with elastography), has gained both theoretical and experimental significance. In this review, McIlvain discusses the importance of the viscous-to-elastic ratio in describing brain function and health.

## Graphical Abstract

**Graphical Abstract:**
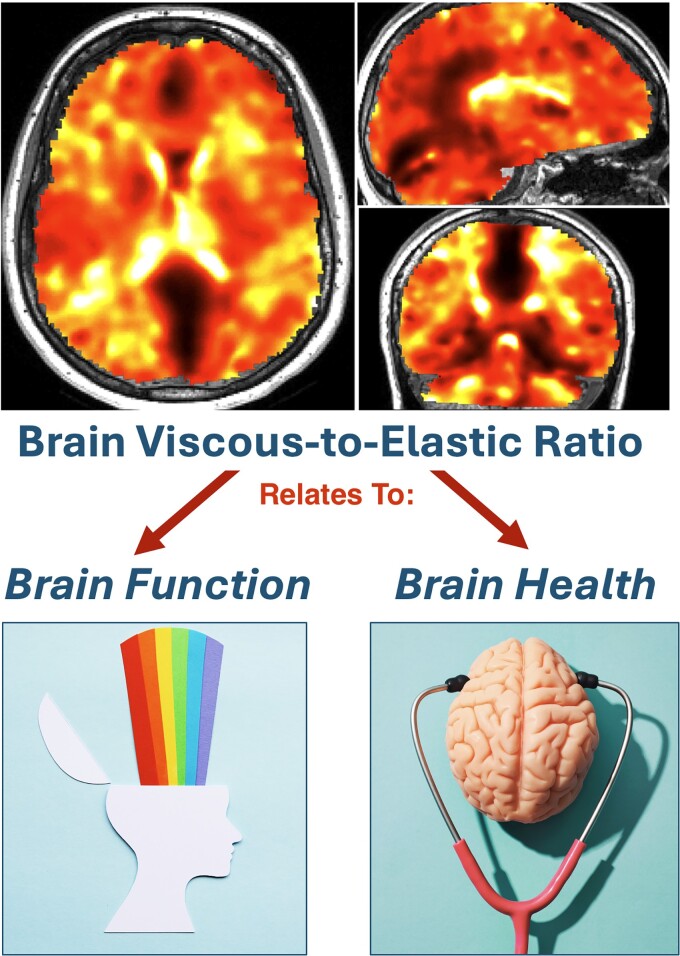

Magnetic resonance elastography (MRE) is an advanced MRI technique that has emerged over the last two decades as a tool for non-invasive measurement of tissue mechanical properties.^1^ MRE produces quantitative maps that sensitively characterize tissue microstructure. Mechanical properties reflect underlying cellularity and describe not only the properties and relative proportions of the individual component materials but also their shape and connectivity at the microscale.^2^ MRE has applications in nearly every soft tissue in the body,^3^ but given the structural intricacies and complex functionality of the brain, a rising interest has been placed on using MRE to study the mechanical properties of neural tissue *in vivo*.^4^

Brain MRE has revealed tissue structural changes during aging,^5^ neurodegenerative disease,^6^ neurodevelopment,^7^ stroke^8^ and traumatic brain injury.^9^ MRE is complementary to standard imaging and captures ‘a unique aspect of brain structure’; when MRE is used in conjunction with other imaging to predict disease, combining imaging modalities outperforms the predictive ability of any measure individually.^10^ Therefore, MRE offers a valuable imaging contrast for characterizing brain tissue, and study structure–function relationships and the microstructural changes from neuropathology.

An MRE exam is conducted using externally generated shear waves at a set frequency to create micron level displacements in brain tissue. These displacements are imaged using phase-contrast MRI and converted to maps of mechanical properties through an inversion algorithm.^11^ MRE was originally developed to measure tissue stiffness, or the ability of a material to resist a deformation, and this remains its primary use. While it is less common, MRE can also be used to calculate other mechanical properties, including the ratio of the tissue loss modulus to tissue storage modulus, or the viscous-to-elastic ratio, which describes the relative ability of a material to dissipate energy under deformation.^12^ This relative mechanical measure has gained experimental significance as the brain MRE field has grown.

We now believe that describing relative tissue viscosity may have meaningful relevance in differentiating normal aging from neurodegeneration, in sensitively measuring individual differences in cognitive function, in understanding how the brain responds to mechanical stress and in differentiating cell types in tumours. However, literature on brain viscosity is limited, and it is often presented peripherally to the stiffness findings. While measures that describe the relative viscosity of tissue are gaining relevance, its potential clinical importance has not been fully actualized. Here, we aim to discuss the relevant literature that uses MRE to measure the viscous-to-elastic ratio of brain tissue, discuss challenges in measuring the mechanical viscosity of tissue and speculate on what it signifies biologically.

## Governing equations of brain viscosity

Brain tissue can be modelled as a viscoelastic material characterized by its time dependent response to deformation.

Under harmonic excitation at frequency *ω*, the motion reaches a steady state where the displacement,u, at each time, *t*, and each point, x, is given by:


(1) u(x,t)=U(x)cos(ωt+P(x))=Re{U*(x)eiωt}

where U(x) is the amplitude of the displacement, *P* is the phase shift, and $U^{*}(x)=U(x)e^{iP}$ is the complex-valued amplitude encompassing both the amplitude and phase of the sinusoidal motion. In purely elastic materials, the applied stress and resulting strain are perfectly in phase, whereas in viscoelastic tissue, there is a phase lag between the applied stress, $\sigma =\sigma_{0}\cos(\omega t)$ and the resulting strain, $\epsilon =\epsilon_{0}\cos(\omega t-\phi_{\epsilon })$. This phase lag, $\phi_{\epsilon }$, measured in radians, indicates the level of damping in the material.

Steady state oscillatory motion in as described by the displacement equation (1) can be induced though low-amplitude vibration and imaged with MRI using motion encoding gradients (*g*), synchronized to the period of vibration. Motion encoding gradients are shifted with respect to the trigger in evenly partial wave increments of $t_{0}$ over a series of partial acquisitions to capture snapshots of wave progression across the harmonic cycle, which can be mapped into the MR phase signal (*ϕ*) as:


(2) $$\phi (x,t_{0})=\;\gamma \overset{\mathrm{TE}}{\underset{0}{\int }}\;u(x,t-t_{0})g(t)\;dt$$

with *γ* as the gyromagnetic ratio of the element of interest. The phase of the complex-valued MRI data, *ϕ*, is converted to maps of the complex-valued displacement vector ($\vec{u^{*}})\;$using a temporal Fourier transform as $\vec{u^{*}}(x)=\;F\{\phi (x,t)\}{|}_{\omega }$.

These measurements are then used to compute maps of mechanical properties, through the inverse solution to Navier’s equation of motion:


(3) $$\nabla \cdot G^{*}(\nabla \vec{u^{*}}+\nabla {\vec{u^{*}\;}}^{T})+\;\nabla \lambda \nabla \cdot \vec{u^{*}}=\;\rho \omega^{2}\vec{u^{*}\;}$$

where G*, is the complex-valued shear modulus of interest for MRE, ρ is density (assumed ∼1000 kg m^−3^), and λ is the first Lamé constant. $G^{*}$ has storage and loss components Gʹ and G″, respectively, as:


(4) $$G^{*}=G^{\prime }+iG^{\prime \prime }$$

For ease of interpretability and clinical translatability, $G^{*}$ is often converted to the more commonly reported measure, shear stiffness, *μ*, which is calculated as $\mu =\frac{2{\left|G^{*}\right|}^{2}}{G^{\prime }+\;|G^{*}|}$. Here, we focus on the viscous-to-elastic ratio, which can similarly be measured from $G^{\prime }$ and $G^{\prime \prime }$. Relative viscous properties of tissue can be reported in several ways:

Damping ratio, ζorξ, is a unitless measure of how quickly mechanical energy is dissipated into thermal energy in a contained system. It is a ratio of the actual damping divided by the critical damping in the system and is defined as: $\zeta \;\mathrm{or}\;\xi =G^{\prime \prime }{/2G}^{\prime }$.
Phase angle, φ, is a related measure that describes the lag between the maximum stress and maximum strain in a viscoelastic material. It is defined as: $\varphi =\arctan(G^{\prime \prime }/G^{\prime })$ and has sometimes been referred to in the literature under the term ‘fluidity’. In some *ex vivo* MRE literature, φ is reported as tan *δ*, where $\tan(\delta )=(G^{\prime \prime }/G^{\prime })$.^13,14^
Alpha, *α*, is used in the context of the spring-pot model and allows a material’s behaviour to be characterized simultaneously in the time and frequency domains. The spring-pot is modelled through the parameter *α*, where:
  $$G^{*}(i\omega )=K(i\omega {)}^{\alpha },$$

with *K* and *α* being the unknown variables.^15^ Alpha is translated into storage and loss modulus through the relationship, $\alpha =\frac{2}{\pi }\arctan\;(\frac{G^{\prime \prime }(\omega )}{G^{\prime }(\omega )})$, by fitting a powerlaw exponential across Gʹ and G″. *α* describes the fit of the curve in response to varying frequency, with the ratio G″/Gʹ being a constant.

All three parameters, damping ratio, phase angle and alpha, represent the tissue’s response under loading, balancing viscous and elastic forces in the tissue. Therefore, we will group these measures together under the terminology ‘viscous-to-elastic ratio’ for the remainder of this review.

## The viscous-to-elastic ratio varies regionally and differently than stiffness

**Figure 1:**
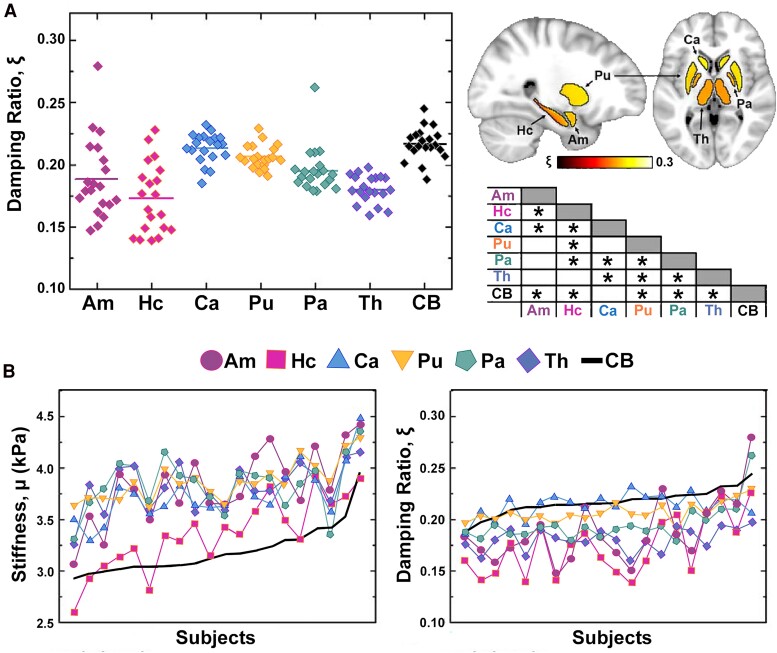
**The viscous-to-elastic ratio of tissue varies regionally and differently than stiffness.** (**A**) Damping ratio measured at 50 Hz in *N* = 20 males age 18–33, in the subcortical grey matter structures (Am, amygdala; Hc, hippocampus; Ca, caudate; Pu, putamen; Pa, pallidum; Th, thalamus) and the cerebrum (CB). Average damping ratio is shown in MNI space. Markers on the stair chart indicate the differences between structures found through *post hoc* paired *t*-test (*P* < 0.05 after Holm–Bonferroni correction). Adapted from Johnson, CL. *et al*. Viscoelasticity of subcortical gray matter structures. *Human Brain Mapping* (2016). © 2016 Wiley Periodicals, Inc.^18^with permission. (**B**) Stiffness and damping ratio of each structure sorted by property value of the cerebrum. Adapted from Johnson, CL. *et al*. Viscoelasticity of subcortical gray matter structures. *Human Brain Mapping* (2016). © 2016 Wiley Periodicals, Inc.^18^ with permission.

The brain is a mechanically heterogenous organ, with small neighbouring substructures having important and varied input to human function. In general, MRE literature reports that cortical grey matter is softer than white matter,^16^ but most studies do not report differences in viscosity between cortical grey and white matter.^17^ Despite lack of global differences, several studies have reported that subregions of grey and white matter show significant differences in relative viscosity between them, sometimes even more strongly than stiffness.^18,19^ For example, the corpus callosum has a 26% lower viscous-to-elastic ratio than average white matter, while its stiffness is 16% higher than white matter.^17^ Subcortical grey matter structures, in general, have a lower viscous-to-elastic ratio than cortical grey matter. The hippocampus shows the largest differences in viscous-to-elastic ratio from the average cerebrum, of 20% lower in young healthy adults (Fig. 1A), this trend is consistent in both children^20,21^ and older adults.^19^ Even hippocampal subfields present as regionally different, with the entorhinal cortex having a 27.6% lower relative viscosity than the dentate gyrus.^22^ The underlying structural patterning that makes average white matter no different in relative viscosity than average cortical grey matter, but the corpus callosum significantly lower than white matter and the subcortical structures lower than cortical grey matter, remains to be understood. It is possible that these effects are biological, and that structural anisotropy, cell density or differences in vasculature contribute to this effect, however it is also possible that the inversion is having an outsizes influence on measured regional differences (see Section 8: The viscous-to-elastic ratio is challenging to measure), and more work remains to understand these effects.

Interestingly, while stiffness has been reported as having high intra-subject correlations between structures (i.e. if you have a more stiff hippocampus, you are also likely to have a more stiff thalamus), Johnson *et al.*^18^ showed a lack of these same intra-subject correlations for relative viscosity (Fig. 1B). Which means unlike stiffness, if an individual has a high relative viscosity in one structure, the same won’t necessarily be the case in the other structures. Tissue viscosity does not appear to be impacted by sex.^5,20,23,24^

## The viscous-to-elastic ratio changes with age

Both stiffness and viscosity have been observed to change regionally with age, though interestingly their age-related patterning is independent of one another. Stiffness has been well defined as decreasing both globally and in nearly all brain regions during normal aging, while relative viscosity appears to increase during aging in some, but not all, regions.

**Figure 2:**
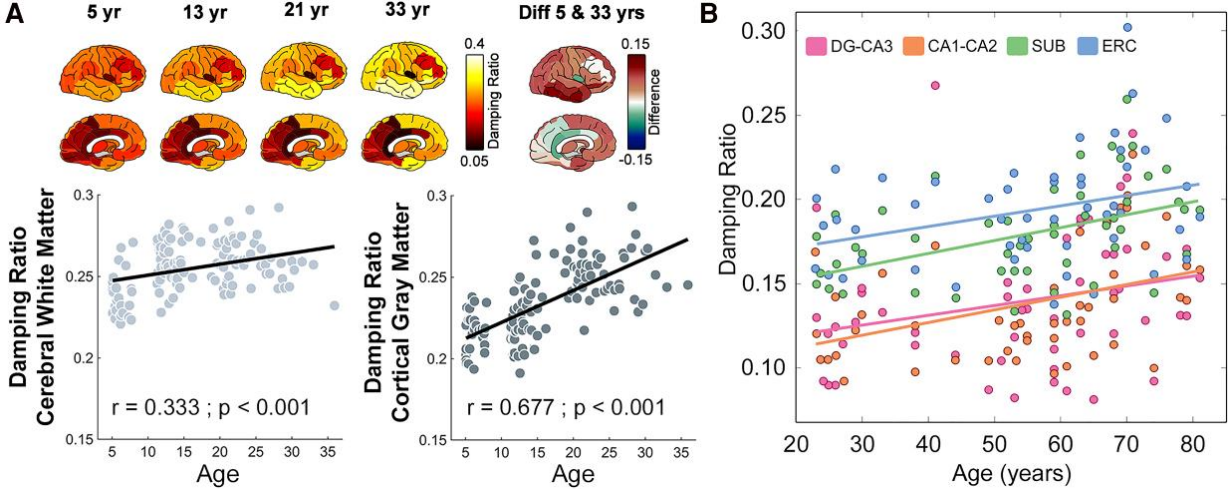
**The viscous-to-elastic ratio changes with age.** (**A**) Study of brain mechanical properties in 125 people ages 5–35 years (yr) old. Images represent regional average brain damping ratio at age 5, age 13, age 21 and age 33 as well as the difference in damping ratio between age 5 and age 33. Graphs show the relationship between damping ratio and age, with damping ratio increasing during maturation more in the cortical grey matter than in the cerebral white matter. After an ANOVA, Tukey *post hoc* tests were used to identify age relationships for each region, with Bonferroni adjustments made for multiple comparisons. Damping ratio was linearly regressed against age separately for each ROI, and correlation coeﬃcients, *r*, were calculated for each relationship of regional measure with age. Adapted from McIlvain, G *et al*. Mapping brain mechanical property maturation from childhood to adulthood. *NeuroImage*, (2022), https://doi.org/10.1016/j.neuroimage.2022.119590.^20^ (**B**) Average damping ratio calculated in each of the subfields of the hippocampus (DG, dentate gyrus; SUB, subiculum; ERC, entorhinal cortex) in 54 people ages 23–81 shows age related increases to damping ratio across adulthood. Reproduced by permission of Oxford University Press from Delgorio, PL., *et al*. Effect of aging on the viscoelastic properties of hippocampal subfields assessed with high-resolution MR elastography. *Cerebral Cortex* (2021).^22^

Unlike stiffness, several studies show that the global average brain does not change considerably in the viscous-to-elastic ratio during aging,^5^ but that individual anatomical regions change considerably during maturation and somewhat during aging. During maturation, both cortical grey matter and white matter increase in relative viscosity, with one study reporting a white matter increases 8.5% and cortical grey matter increases 27.2% between age 5 and 35^20^ (Fig. 2A), and a second showing ∼8% white matter increases between age 7 and 17.^25^ In both studies, the percentage of change in relative viscosity over this timeframe was nearly double that of changes to white matter stiffness.

In aging, regional changes to relative viscosity are apparent in some subcortical grey matter structures. For example, the relative viscosity of the hippocampus appears to change considerably with aging, being nearly 21% higher in adults aged 70 than adults aged 25.^19^ Even the hippocampal subfields follow detectability different trajectories with age, though all are increasing^22^ (Fig. 2B). The cellular basis behind why some sub structures change more in relative viscosity during maturation and aging is understudied, but the existence of these differences emphasizes the need for both stiffness and relative viscosity to be measured when reporting age-related brain mechanical properties.

## The viscous-to-elastic ratio is a clinically relevant measure

### Neurodegenerative disorders

Neurodegenerative disorders are characterized by neuropathologic changes, which lead to a variety of clinical symptoms, including cognitive deficits. Other imaging techniques have revealed this pathology, but tissue mechanical properties have emerged as a potentially more sensitive non-invasive metric for describing underlying microstructural changes.^6^ Brain stiffness has been observed to decline in people with multiple sclerosis,^26^ Parkinson’s disease,^27,28^ Alzheimer’s disease^29,30^ and stroke.^8^ The viscous-to-elastic parameter has not yet been comprehensively evaluated in these disease states, but existing work appears to demonstrate that viscosity reveals pathologic trends that are separate from and complementary to those found by measuring tissue stiffness.

#### Demyelination

**Figure 3:**
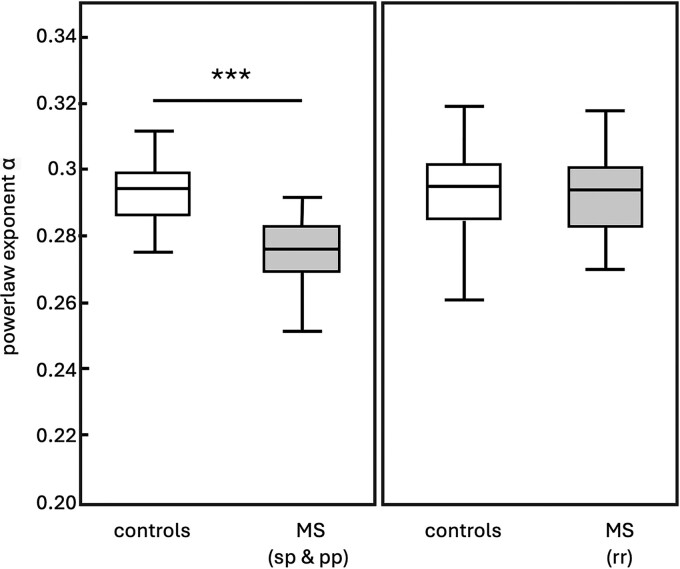
**The viscous-to-elastic ratio is a clinically relevant measure for evaluating multiple sclerosis.** Patients with secondary progressive (sp; *N* = 17) and primary progressive (pp; *N* = 6) forms of multiple sclerosis show significantly reduced powerlaw exponent alpha values (6.07%; *P* < 0.001) compared to healthy controls (*N* = 38), while people with relapsing remitting (rr; *N* = 45) multiple sclerosis do not show differences. Differences between patients and controls regarding age were analysed by the Mann–Whitney U-test. Reproduced with permission from Streitberger, KJ, *et al*.^32^

Myelin facilitates efficient transmission of impulses along a neuron and is a critical component of normal brain function, and diseases like multiple sclerosis cause a rapid destruction of myelin.^31^ While both severe and mild forms of multiple sclerosis show significant decrease in stiffness, decreases to viscosity have only been observed with in people with chronic-progressive of multiple sclerosis, and not in the milder forms of the disease including relapsing remitting multiple sclerosis^26,32^ and clinically isolated syndrome^33^ (Fig. 3). We hypothesize that acute multiple sclerosis causes inflammation and loss of myelin integrity, without meaningful loss to the overall tissue mechanical matrix integrity, thereby changing stiffness, but not relative viscosity, whereas severe multiple sclerosis produces a complete loss of myelin and neurons, thereby altering both tissue composition and organization, which is reflected by changes to both stiffness and viscosity.^34^

Changes to the viscous-to-elastic ratio appear to be proportional to loss of quantity of myelin. A mouse model treated with cuprizone to induce demyelination showed progressive decrease to the tissue’s relative viscosity with treatment period, and this decrease was directly related to the post-mortem myelin counts.^35^ Notably, because myelin recovery can occur after cessation of cuprizone treatments, it was also observed that remyelination was related to recovery of relative viscosity. This finding not only highlights the sensitivity of the viscous-to-elastic ratio but also endorses that mechanical property changes from damage are not permanent; therefore, MRE can be used as a sensitive tool for tracking tissue response to disease treatment.

It can be clinically challenging to know when multiple sclerosis has crossed from the acute phase to the chronic phase, knowing that both stiffness and viscosity are affected in chronic multiple sclerosis, but only stiffness is affected in acute multiple sclerosis, may be a valuable clinical indicator for monitoring disease progression. Other demyelinating disorders have not yet been studied using MRE, but diseases including neuromyelitis optica, anti-NMDA MOGAD, and Lyme’s disease cause demyelination, and observation of acute and chronic changes to brain mechanical properties may inform care in these conditions.

#### Neurons loss

Several types of idiopathic dementia show more loss of neurons, including Alzheimer’s disease, where beta-amyloid plaques and tau protein tangles disrupt cell communication and lead to their death,^36^ and Parkinson’s disease that is characterized by a loss of dopamine producing neurons.^37^

Alzheimer’s disease shows significantly reduced brain stiffness and relative viscosity compared to controls,^29,38^ with relative viscosity being most reduced in the global white matter, the hippocampus and the thalamus.^38^ However, relative viscosity is less sensitive to the disease than other measures, as it is only able to predict Alzheimer’s disease with an accuracy of 79%, comparable to prediction capability of hippocampal stiffness at 81%, volume at 86% and mean diffusivity at 83%.^38^ But, notably, when these four structural measures were combined, prediction capabilities increased to 90%, though it is unclear the unique contributions of viscosity over stiffness in prediction of Alzheimer’s disease state.

A particularly interesting area for further consideration is the heightened sensitivity of viscosity to ‘early’ dementia changes compared to stiffness. An animal study on Alzheimer’s demonstrated that it is possible to observe changes to relative hippocampal viscosity prior to onset of clinical symptoms, but this same thing was not true for stiffness.^39^ Similarly, in people with mild cognitive impairment, relative hippocampal viscosity differences from controls had a stronger effect size than hippocampal stiffness differences from controls.^40^ The potentially unique sensitivity of relative hippocampal viscosity for early Alzheimer’s disease changes requires more comprehensive investigation.

While brain mechanical properties in Parkinson’s disease have been less studied, Parkinson’s disease appears to result in similar loss to relative tissue viscosity. In Parkinson’s disease, up to 8% decreases have been found in the relative viscosity of the lentiform nucleus, a region involved in regulation of motion, while stiffness in the same region is just 1.3% less than controls.^28,41^ Similar decreases to relative viscosity have also been observed in the lentiform nucleus in people with progressive supranuclear palsy (PSP), a clinically similar disease to Parkinson’s disease. Interestingly in PSP, unlike in Parkinson’s disease, stiffness was reduced by nearly 38%. PSP also saw decreases in relative viscosity in the striatum region, where these changes were not found in Parkinson’s disease.^41^ While PSP shows similar movement and memory impairments to Parkinson’s disease, their underlying pathology are distinctly different. Parkinson’s disease arises from a localized deposit of alpha-synuclein proteins in the substantia nigra, while PSP arises from a non-localized deposit of tau proteins.^42,43^ PET imaging can differentiate locations of protein deposits, but is ill-suited for routine clinical screening, compared to safer methods like MRI, making these observed mechanical property differences of high clinical value.

The utility of MRE to assess Alzheimer’s disease and Parkinson’s disease is only beginning to fully actualize. The imaging quality, resolution and repeatability of brain MRE are rapidly expanding, and many of these studies were conducted before advanced imaging methods were developed. Utilizing improved methods, the viscous-to-elastic ratio has potential to become a clinically important metric in neurodegenerative disease. Some diseases that result in a loss of neurons and have not yet been studied with MRE including Huntington’s disease, Tay–Sachs disease, Niemann–Pick disease and infections such as encephalitis and meningitis, while others have been studied only for brain stiffness and not yet for relative viscosity, such as amyotrophic lateral sclerosis.^44^

#### Normal pressure hydrocephalus

**Figure 4:**
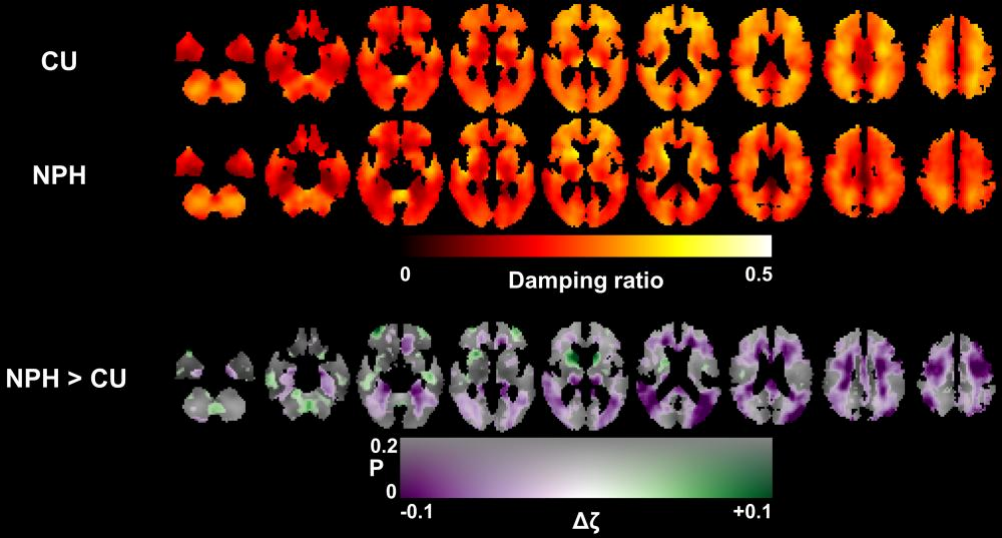
**The viscous-to-elastic ratio is a clinically relevant measure for evaluating normal pressure hydrocephalus.** Average damping ratio maps for cognitive unimpaired (CU; *N* = 44) control subjects, subjects with normal pressure hydrocephalus (NPH; *N* = 33), and group wise differences created using the neural network inversion. Reproduced with permission from Murphy, Matthew C., *et al*. Identification of normal pressure hydrocephalus by disease-specific patterns of brain stiffness and damping ratio. Investigative radiology (2020).^46^

Normal pressure hydrocephalus (NPH), a condition in which excess cerebral spinal fluid is retained in the brain, for largely unknown reasons. This fluid build-up causes displacement of brain tissue and can lead to cognitive and motor symptoms if left untreated. Freimann *et al.*^45^ showed that NPH results in a significant decrease in relative brain viscosity and finds a partial return of relative viscosity with shunt treatment, further indicating that damage to tissue viscosity may be reversible in some cases. Murphy *et al.*^46^ later localized changes to the viscous-to-elastic ratio in NPH to primarily above the level of the lateral ventricles (Fig. 4).

Interestingly in each of these cases of neurodegenerative disease, the viscous-to-elastic ratio decreases in the case of pathology, while many studies of healthy adults, relative viscosity increases with age. This is despite brain stiffness (elasticity) decreasing in both healthy aging and neurodegeneration. This may signify that there are underlying mechanistically different processes affecting tissue viscosity that occur during normal aging and supports prior hypotheses that neurodegenerative disorders are not just an acceleration of the normal aging process. Research shows that viscosity is not significantly different in people with epilepsy,^10^ or between people of differing body mass indexes.^47,48^ While these findings offer MRE as a novel pathway for disease investigation, more work is required to understand the mechanisms of disease that alter relative tissue viscosity.

### Tumours

Brain tumours have markedly different cellularity than normal brain tissue and therefore the viscous-to-elastic ratio of tumours cannot be considered in the context of normal development, aging or even neurodegenerative pathology. Tumours are the most frequently assessed neurological condition in MRE, and it is expected that their relative viscosity may reflect a unique aspect of tumour biological attributes.^49,50^

**Figure 5:**
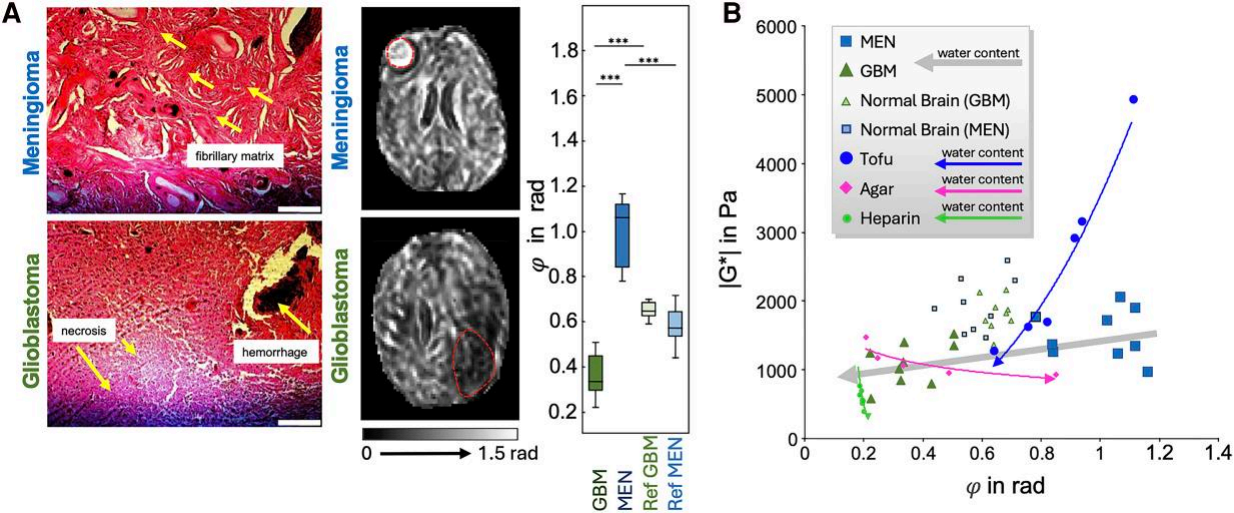
**The viscous-to-elastic ratio is a clinically relevant measure for evaluating brain tumours.** Tumour subtypes show differences in the viscous-to-elastic ratio. (**A**) Histological images (H&E stains) of glioblastoma (GBM; *N* = 9) and meningioma (MEN; *N* = 9) demonstrate fibrillary structures in MEN and necrotic regions in GBM (scale bar: 200 μm). *In vivo* MRE of brain tumours. Two representative cases of MRE in GBM and MEN illustrating differences in phase angle of the shear modulus (*ϕ*) in radians (rad). Dashed lines demarcate tumour regions. Group statistical plot of *ϕ* with differences between tumour entities and corresponding healthy tissue analysed with a two-tailed unpaired Student’s *t*-test. *P* < 0.01 was considered statistically significant. Reproduced with permission from Streitberger KJ, *et al*. How tissue fluidity influences brain tumor progression. *Proc Natl Acad Sci* (2020).^51^ (**B**) Magnitude shear modulus (|G*|) versus phase angle of the shear modulus (*ϕ*) of all tumour values and reference tissues superimposed on phantom data. Possible correlations between viscoelastic properties and water signal were tested using Pearson’s linear correlation analysis. Reproduced with permission from Streitberger KJ, *et al*. How tissue fluidity influences brain tumor progression. *Proc Natl Acad Sci* (2020).^51^

Different types of brain tumours have different mechanical properties both from one another,^49-51^ and from their surrounding tissue.^52^ Gliomas are softer with a lower relative viscosity than healthy tissue, whereas meningiomas are stiffer than healthy tissue,^53-56^ signifying differences in tumour cellular composition.^57^ Histologically, meningiomas have higher neuronal density and organizational anisotropy than both healthy tissue and gliomas,^51^ potentially contributing to their increased relative viscosity when compared to gliomas. Streitberger *et al.*^51^ point to tumour viscosity as having an outside effect on tumour progression (Fig. 5).

The viscous-to-elastic ratio relates to tumour severity, with animal models of gliomas showing a 22% decrease in relative viscosity over just a four-week period of tumour growth.^53^ A second study showed that these declines in viscosity can be slowed or potentially entirely reversed, as mice with glioblastomas, who were treated with B20 anti-VEGF-antibody, showed a higher relative tissue viscosity at all-time points than mice left untreated.^52^ Understanding how treatment impacts tumour mechanics is a nascent but growing area of research.

Understanding tumour mechanical heterogeneity is also of considerable interest for planning surgical resection and broader intervention. Mechanical heterogeneity has been observed on an *ad hoc* basis, such as Schregel *et al.*^53^ who demonstrated a single animal with two distinct tumour sections, with one having more densely packed tumour cells than the other and found differences in stiffness but not in viscosity between the two parts.

Finally, it appears possible to use MRE to study infiltrating brain tumour margins by measuring tumour slip boundaries, as octahedral shear strain measured with MRE shows low strain at the margins with high slip coefficents.^58^ Tumours with high slip are considered self-contained and generally non-infiltrating.

Further study of how the relative viscosity of brain tumours can noninvasively reveal clinically important characteristics is needed, including relating relative tumour viscosity to its molecular profiles, which has been done for tumour stiffness but not viscosity.^59^ There may also exist sizeable potential to understand tumour malignancy with MRE. In a study of breast tumours, relative tissue viscosity was able to differentiate benign and malignant breast lesions,^60^ but these same analyses have not yet been conducted for brain tumours.

## The brain viscous-to-elastic ratio reflects individual differences in healthy individuals

### Cognitive function

**Figure 6:**
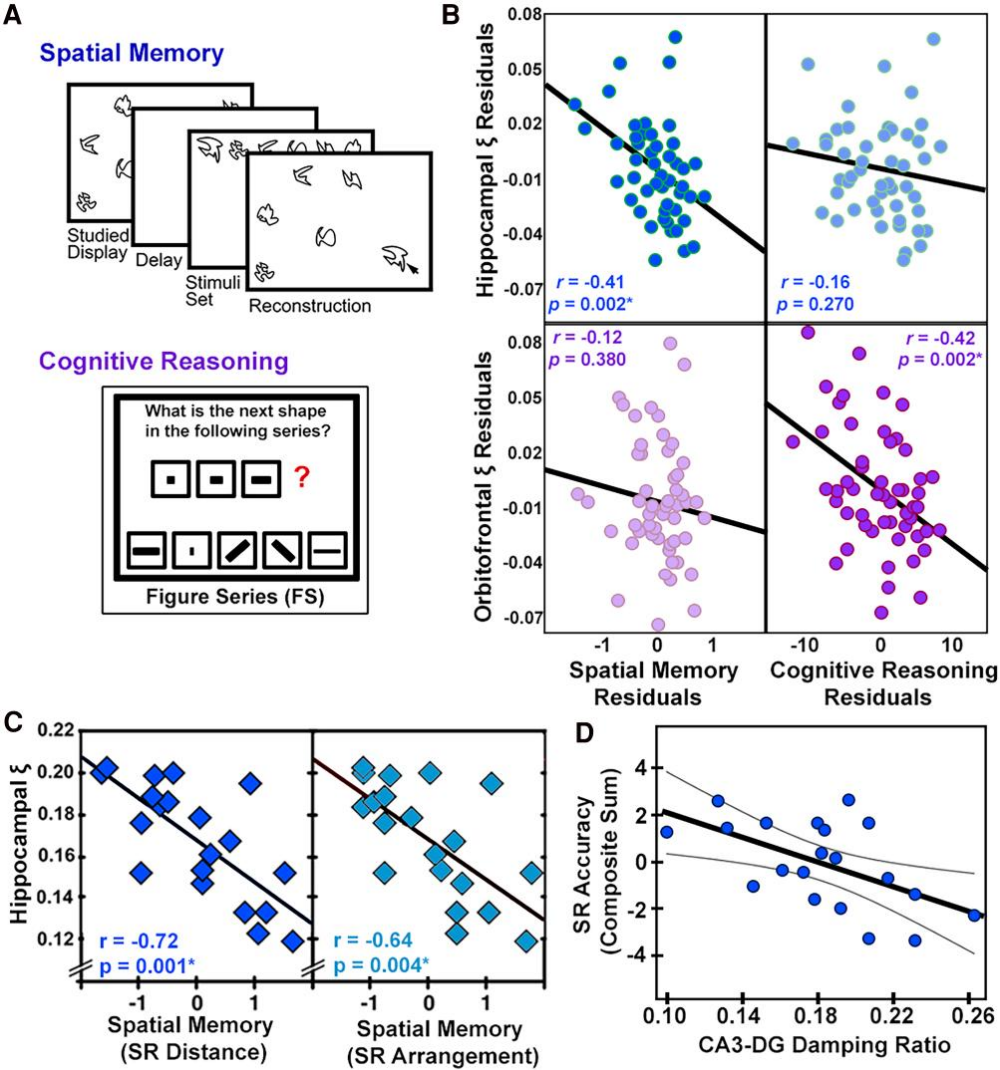
**The brain viscous-to-elastic ratio reflects individual differences in cognitive function in healthy individuals.** Cognitive tasks performance relates to decreased viscosity relative to elasticity. (**A**) Two tasks, a spatial memory task and a cognitive reasoning task have been used to study structure–function relationships with elastography. (**B**) Hippocampal damping ratio relates to spatial memory in *N* = 20 adults, as assessed by calculating the Pearson partial correlation coefficient, *r*, with age and education as control variables. Figure adapted with permission from Schwarb, H. *et al*. Medial temporal lobe viscoelasticity and relational memory performance. *Neuroimage*, (2016) https://doi.org/10.1016/j.neuroimage.2017.10.00862. (**C**) A study done with 53 adults aged 18–35 shows that hippocampal damping ratio residuals relate to spatial memory but not to cognitive reasoning, while orbitofrontal damping ratio residuals relate to cognitive reasoning but not spatial memory, an expected finding based on the known structure–function relationships in the brain. Pearson partial correlation coefficients, *r*, and associated *P*-values are reported, controlling for sex. The significance of correlations was determined at *P* < 0.05 with Bonferroni correction for multiple comparisons. Figure adapted from Johnson, Curtis L., *et al*. Double dissociation of structure–function relationships in memory and fluid intelligence observed with magnetic resonance elastography. *Neuroimage* (2018) https://doi.org/10.1016/j.neuroimage.2018.01.00761, with permission from Elsevier. (**D**) The association between spatial reconstruction accuracy and adjusted damping ratio in the CA3-DG in *N* = 20 healthy young adults. Greater viscoelasticity of the CA3-DG (dentate gyrus) predicted better relational memory accuracy. Adapted with permission from Daugherty, Ana M., *et al*.^64^ ©2020 Massachusetts Institute of Technology. All rights reserved.

Potentially, the most unique aspect of relative viscosity that sets it apart from measures of brain stiffness is the sensitivity of correlations between regional viscosity and cognitive performance. In healthy young adults, relative hippocampal viscosity shows strong correlations with memory performance, including spatial memory (*r* = 0.72),^61-64^ declarative memory (*r* = 0.40)^65^ and verbal memory (*r* = 0.77)^66^ (Fig. 6).

Using MRE, it is even possible to determine in which hippocampal subfield viscosity most strongly relates to task performance, with one study showing that spatial memory is most strongly related to the relative viscosity of the CA3-DG, a finding that makes sense in the context of what is known about the functionality of each subfield^64^ (Fig. 6D) While the hippocampus is overwhelmingly the most studied region in relation to cognitive function, orbitofrontal cortex relative viscosity has also been found to be related to performance on a figure series task,^61^ and ventromedial prefrontal cortex relative viscosity is related to the accuracy of declarative memory.^62^ In each of these studies, a lower relative viscosity, which is consider more elastic tissue, is related to better task performance.

Interestingly, in each of these studies, no significant relationship was found between memory and either hippocampal stiffness or volume. Hippocampal volume has been a long-standing metric of interest in memory performance, as it has been a hallmark of cognitive decline in neurodegenerative diseases like Alzheimer’s.^67^ However, in healthy young adults, hippocampal volume rarely shows the same strong relationships to memory, and during maturation, sometimes even shows the inverse trend.^68^ This highlights that before any mass-atrophy events occur, volumetric measures are fundamentally not well suited to characterize individual differences to underlying cellularity that result in small differences in memory and other cognitive function.

Conversely, mechanical properties are sensitive to underlying cellularity in that they reflect both the density of cells and the degree of interconnectedness of the glial matrix.^69^ This sensitivity of mechanical properties to cognitive function has been demonstrated several times. Using a double dissociation technique, it was found that for two given aspects of cognitive function (in this case spatial memory and fluid intelligence) each ‘only’ related to the relative viscosity of the region known to relate to that function (in this case the hippocampus and orbitofrontal cortex, respectively) and did not relate to the relative viscosity of the other region^61^ (Fig. 6C). Another study demonstrates that performance on the verbal paired associates’ task related to the viscous-to-elastic ratio of the left hippocampus, but not the right hippocampus,^66^ important as the left hippocampus is implicated in verbal memory. In nearly all studies that have been conducted, relative viscosity has related to cognitive function either more strongly or exclusively compared to stiffness. While the reason for this remains unclear, it points to a unique contribution of the viscous-to-elastic ratio measure to describe aspects of underlying cellularity between healthy individuals that warrants more discussion and investigation.

Paediatric structure–function relationships are of notable importance as they provide insight in understanding neurodevelopment encompassing attributes from intelligence to personality, to mental health. One study showed that the relative viscosity of the right inferior frontal gyrus, an area implicated in language, was significantly correlated with vocabulary performance in children ages 5–7 years, while the same relationship was not found with stiffness.^70^ The exclusivity of correlations between tasks and relative viscosity of brain regions, both during development and in young adulthood, highlights the sensitivity of regional brain viscosity and underscores a need to expand this work.

### Aerobic fitness

**Figure 7:**
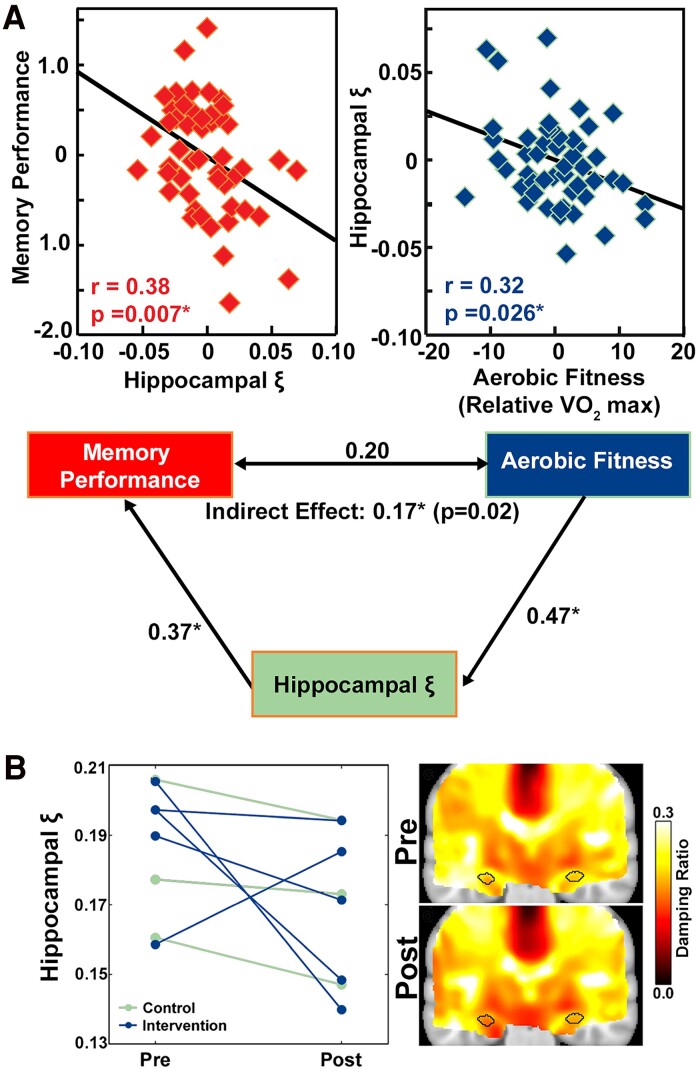
**The brain viscous-to-elastic ratio reflects individual differences in aerobic fitness in healthy individuals.** (**A**) Hippocampal damping ratio residuals plotted against spatial reconstruction memory task residuals and VO2max aerobic fitness score residuals in *N* = 51 healthy young adults aged 18–35. Pearson partial correlation coefficients, *r*, with age and sex as control variables were used, with *P* < 0.05 as statistically significant. Mediation model testing the effect of aerobic fitness on relational memory mediated by hippocampal damping ratio. A path model included age and sex as covariates to each measure and non-significant paths were constrained in the final model. Adapted from Schwarb, H, *et al*. Aerobic fitness, hippocampal viscoelasticity, and relational memory performance. *Neuroimage* (2017)^63^  https://doi.org/10.1016/j.neuroimage.2017.03.061 with permission from Elsevier. (**B**) Plots and average property maps of hippocampus damping ratio for individuals (*N* = 8) in both groups pre- and post-treadmill walking exercise training intervention showing a lower relative viscosity of 11.5% decrease with a Cohen’s d effect size of −1.20. Adapted with permission from Springer Nature from Sandroff, BM. *et al*. Exercise training effects on memory and hippocampal viscoelasticity in multiple sclerosis: a novel application of magnetic resonance elastography, *Neuroradiology* (2017).^72^

Cognitive function is demonstrated to be linked to aerobic exercise, as exercise is expected to improve brain health.^71^ Recently, it has been found that brain mechanical properties, including both stiffness and relative viscosity, are integral to this relationship. Schwarb *et al.*^63^ showed that in healthy young adults, relative hippocampal viscosity related to both individual’s VO_2_ maximum (*r* = 0.32) and memory (*r* = 0.38), while VO_2_ and memory were not significantly correlated with each other (*r* = 0.09) (Fig. 7A). By developing a mediation model, Schwarb *et al.*^63^ showed that relative viscosity was a key mediator in the indirect relationship between aerobic fitness and memory performance, with a lower relative brain viscosity indicative of better performance. Support for the relationship between hippocampal viscosity and fitness was strengthened by Sandroff *et al.*,^72^ who showed a 11.5% decrease in the relative viscosity of the hippocampus before and after a 12-week fitness intervention in people with multiple sclerosis and had a higher effect size than the exercise related changes to stiffness with an increase of 8.4% (Fig. 7B). More work is needed in this area to characterize how relative tissue viscosity may mediate cognitive function and aerobic fitness.

Relatedly, a significant effort has been put forward to understand how blood flow affects measured mechanical properties. One study found that when passively measured, the differences in cerebral blood flow (CBF) between subcortical structures were not reflected by relative viscosity,^73^ but when CO_2_ was administered to increases CBF, small but significant increases to relative viscosity were found.^74^ Interestingly, a separate study showed that during the Valsalva manoeuvre, participants had significant decreases in relative brain viscosity.^75^ CO_2_ administration is known to cause vasodilation (increasing CBF), while the Valsalva manoeuvre temporarily decreases CBF, therefore inverse responses of relative viscosity under these two conditions lend support to the idea that the viscous-to-elastic ratio is linked, at least in part, to cerebral haemodynamics. Likewise, acute exercise has been shown to significantly increase to relative tissue viscosity immediately after exercise by ∼5%, and return to baseline 1 h later, a change that was attributed to blood flow changes during intense exercise.^76^ Finally, study showed a slightly higher relative viscosity during systole than diastole,^77^ but Hannum *et al.*^78^ demonstrated that these changes could be the effect of cardiac noise during the motion encoding. It appears likely that blood flow accounts for a portion of acute changes to relative tissue viscosity, but more work is in this area will improve our understanding of this phenomenon.

## The viscous-to-elastic ratio is thought to reflect tissue organization

While measuring relative brain tissue viscosity appears to be valuable in differentiating aspects of neurodegeneration, assessing tumour characteristics, and sensitively measuring individual differences in cognition and fitness, the question of what tissue viscosity is reflecting at the cellularly level is ill defined, and has mainly been left to speculation on a population specific basis.

**Figure 8:**
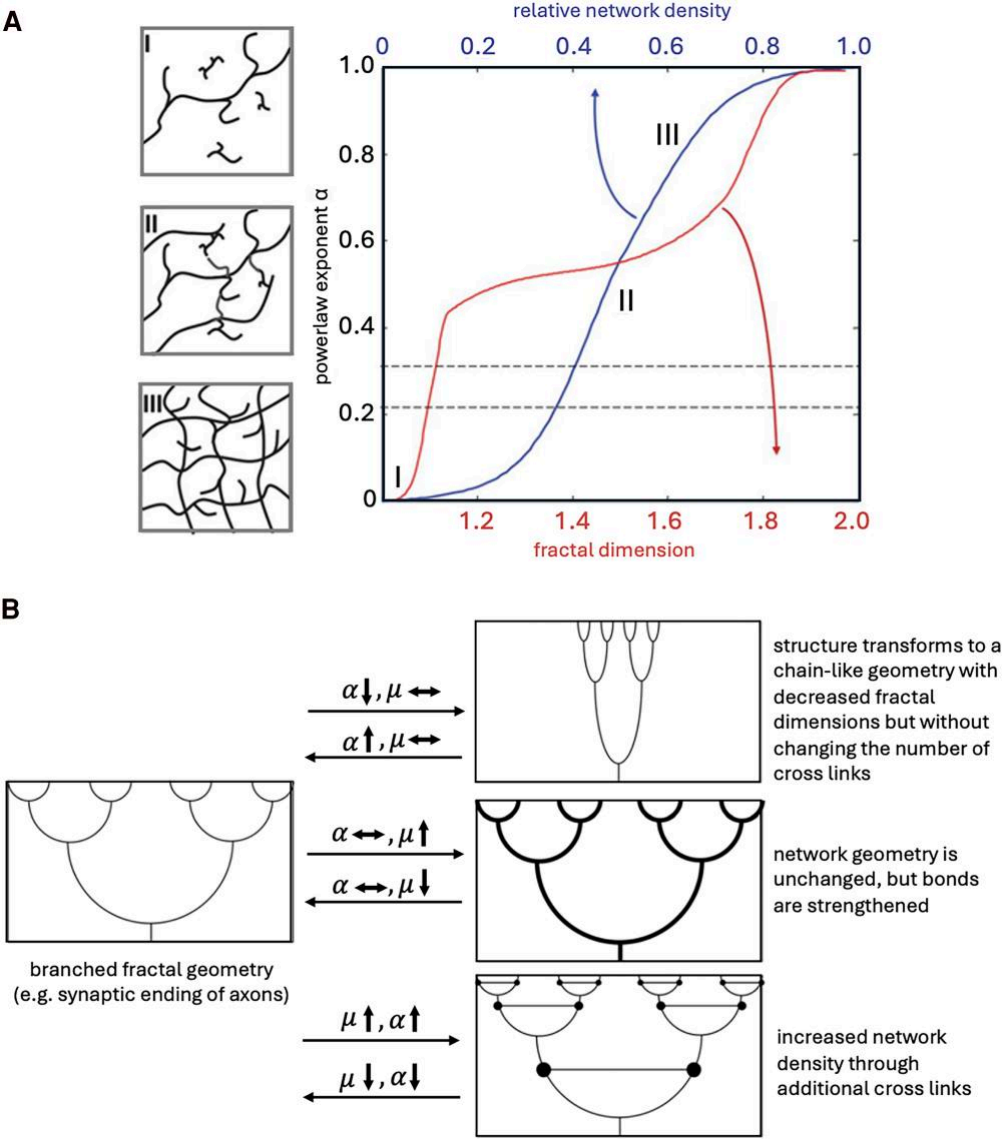
**The viscous-to-elastic ratio is thought to reflect tissue organization**. (**A**) Simulation of the effective viscoelastic response in 2D-mesh parameterized by the powerlaw exponent, *α*. Reproduced with permission of Royal Society of Chemistry, from Sack I, *et al.*,^81^ permission conveyed through Copyright Clearance Center, Inc. (**B**) Schematic of the changes in principal structures in mechanical lattices and their theoretical effects on shear stiffness, *µ*, and powerlaw exponent, *α*. Reproduced with permission of Royal Society of Chemistry, from Sack I, *et al.*,^81^ permission conveyed through Copyright Clearance Center, Inc.

Early work by Posnanski *et al.*^79^ and Guo *et al.*^80^ proposed a fractal model to describe the viscous behaviour of brain tissue, describing tissue as a series of cross-linked viscoelastic points embedded into a non-compressible background. Using simple experiments, they showed that G″, was sensitive to increases in fractal dimension, but G′ was not. Following that work, Sack *et al.*^81^ expanded the model to describe a fractal geometry in which the tissue can (i) increase or decrease in density; (ii) grow to have stronger or weaker bonds; or (iii) transform to have a larger or smaller fractal dimension (Fig. 8). Under these groupings, the relative viscosity of the tissue would only be affected by the first and third scenarios, while stiffness would only be affected by the second and third scenario. Broadly these studies describe the notion that the viscous-to-elastic ratio is associated with the ‘organization’ and ‘connectedness’ of tissue, as opposed to stiffness, which has long been thought to be associated with the ‘composition’ of tissue.

The brain is comprised of neurons and glial cells.^82^ Cells including astrocytes and fibroblasts are elongated fibrous cells with a relatively stiff cytoskeleton, whereas microglia are rounder and more amorphous in structure.^83^ The structure of individual cells in the aggregate contributes to overall changes to tissue mechanical properties. Cells that have elongated and defined structure are theorized to contribute to higher stiffness and lower relative viscosity, whereas cells with amorphous structure are theorized to contribute to lower stiffness and higher relative viscosity.^84,85^ In addition to individual cells, tissue mechanical properties are affected by cell-cell interactions.^85^ One oligodendrocyte provides the myelin sheaths to multiple neurons, thereby increasing matrix connectedness.^86^ Similarly, astrocytes link neurons to improve chemical signalling.^87^

On the molecular level, one study assessed mice during development and provided histology and proteomic measures,^88^ finding relative tissue viscosity was sensitive to decreases in structural plasticity and cell adhesion proteins including the Robo2 and ECOM protein and the DPYSL protein family, which are related to actin crosslink formation.^88^ Such that decreases in cell adhesion and crosslink proteins are related to decreases in viscosity when compared to elasticity. They also demonstrated that the typical decrease to hippocampal neurogenesis found during development was related to a decrease in relative tissue viscosity. Both pieces of evidence from this study experimentally provide evidence that the viscous-to-elastic ratio is closely linked to tissue organization.

## The viscous-to-elastic ratio is contradictory in directionality of improvement

The relative viscosity of tissue is expected to be influenced by the organization and connectedness of the glial matrix, which are affected by development, aging and pathology in different ways.

In healthy young adults, better cognitive function is associated with decreased relative viscosity, which is indicative of a well-connected and organized elastic tissue. As people age, neuronal loss is inevitable, resulting in a loss of stiffness. However, cell death alone is not expected to be the driving factor in changes to relative viscosity, as evidence by Freimann *et al.*^8^ who showed that decreased neuronal counts changed stiffness but not the viscous-to-elastic ratio. The ‘disconnection hypothesis’ says that during the normal aging, the brain ‘loses network interconnectedness’.^89,90^ Loss of white matter anisotropy,^91^ declines to oligodendrocyte efficiency and degradation of myelin integrity have all been observed during normal aging. Some studies show that the number of oligodendrocytes actually increases with age,^92^ but are more fragile, produce thinner myelin sheaths and myelinate less thick neuronal axes.^93^ This loss of matrix integrity and interconnectedness, without substantial loss in cell density, results in tissue with high levels of sub-optimally connected matter, leading to ‘higher’ relative viscosity in older adults.

In neurodegeneration however, different biological mechanisms cause tissue degradation and decreases to relative tissue viscosity. For example, plaques and neurofibrillary tangles are hallmarks of several degrative diseases and their accumulation around the neuron may alter the mechanical integrity of the glial matrix.^94^ In people with multiple sclerosis, the number of oligodendrocytes declines, resulting in build-up of unattached myelin,^95,96^ which is potentially decreasing the relative viscosity of the tissue. Interestingly a number of studies have shown that inflammation alone does not change the brain’s relative viscosity.^97-99^ Likewise, acute multiple sclerosis, which is primarily inflammation does not result in changes to relative viscosity, while chronic multiple sclerosis, which results in cell death, does change relative viscosity. Overall, the mechanisms driving changing relative viscosity may be the results of a complex interaction of various phenomenon, and more research is needed to parse these mechanisms.

## The viscous-to-elastic ratio is challenging to measure

A major challenge in interpreting the viscous-to-elastic ratio of brain tissue is that it has historically been hard to measure. Brain tissue is fundamentally more lossless than lossy,^100^ making G″ more susceptible to noise. While improvements have been made in the last several years to minimize these effects, older methods may have contributed to inconsistent results across studies. Further, the methods for describing relative brain viscosity are varied between research groups, as discussed earlier, *ξ*, φ and *α* are similar, but not identical, measures.

There is a lack of consensus about which frequency of vibration to use for brain MRE, with 50 Hz or 60 Hz being common, but in practice, a range of frequencies between 10 and 100 Hz can sample the brain tissue.^101^ Longer waves propagate further, but provide less specific information, as their wavelengths span a greater area, and shorter waves dissipate more quickly. These changes to wave behaviour may affect G′ and G″ differently, making the viscous-to-elastic ratio a frequency dependent parameter.^102^ For example, two separate reports (one about maturation, one about aging) show significant age-related changes to the loss modulus at 60 Hz but fails to find significance at 40 Hz or 80 Hz.^24,25^ This may indicate a need for multifrequency approaches, which adds significant time to the imaging, but could more comprehensively describe tissue mechanical behaviour.^103^

Wave inversion technique can affect measured brain viscosity.^104,105^ Direct inversions (DI)^106,107^ primarily include multifrequency dual elasto-visco inversion,^108^ and a heterogeneous multifrequency direct inversion),^109^ as well as several other, mostly outdated, 2D inversion methods. Another inversion option developed specifically for the brain is the nonlinear inversion (NLI),^110^ which uses a finite element model to discretize the brain and iteratively minimize an objective function.^101^ Most recently, a novel approach of using an artificial neural network (NNI) to perform inversions has been developed.^111^ Each of these inversion methods has the same objectives but require slightly different assumptions and has different sensitivity levels, thereby making measured relative viscosity somewhat dependent on which inversion method was used to calculate it.

The major challenge with MRE inversions is many of them have only been systematically validated for measuring stiffness.^109,112,113^ Even when a measure of relative tissue viscosity is reported, it is not always done so under optimal conditions, for example, one study^108^ validated multifrequency inversion using an agarose phantom, which had a loss modulus 10-fold smaller than of the brain.^114^ However, there are ongoing efforts to improve this problem. For instance, Murphy *et al.*^111^ validated NNI for measuring the viscous-to-elastic relationship by demonstrating that it is possible to change the recovered values of relative viscosity without changing values of stiffness.^46,104^ Solamen *et al.*^115^ showed using phantoms made tofu that the relative viscosity of inclusions as small as 8 mm diameter can be successfully identified using NLI. And, Williams *et al.*^116^ have developed a novel phantom that uses LPAA embedded in a PAA background, through which, varying LPAA concentration changes viscous-to-elastic ratio but does not change stiffness.

## Future lines of study and consideration

There is still much to learn about the biological importance and cellular underpinnings of measuring relative brain tissue viscosity. To begin this type of work, longitudinal studies across the lifespan are necessary along with comprehensive cognitive testing. While lower relative viscosity appears to be an asset in young adult cognitive function, and relative viscosity increases with age, the interaction between aging, relative brain viscosity and cognitive function is not well understood.

Animal models of both healthy tissue and neurodegenerative disease are indispensable for providing insight to the cellular underpinnings of tissue viscosity changes. Understand specifically what types of cellular changes affect viscosity separately from those which affect volume, diffusion, and even stiffness, will allow observed viscosity changes to be a more clinically relevant measure. Animal models will also aid in characterizing tumour viscosity and determining how tumour margins can be delineated, how tumour heterogeneity is assessed, and how viscosity contributes to the ability to stage tumours.

Although the ability to accurately measure relative tissue viscosity has improved significantly over the last several years, there are still progresses to be made. Standardized phantoms that have tuneable viscous properties are hard to make at large scale but will become increasingly necessary to allow serious pursuit of for clinically relevant questions. Furthermore, a comprehensive analysis of relative tissue viscosity over a range of frequencies and using multifrequency MRE is necessary to illuminate best practices for measurement of tissue viscosity.

In summary, brain mechanical properties have emerged as a metric for measuring unique aspects of tissue microstructural integrity. Brain viscosity has historically been a side measure to stiffness, but new evidence of the importance of viscosity is rapidly emerging. Viscosity is now demonstrated to be a clinically important, informationally unique metric, that shows great promise in adding to our understanding of disease and cognitive function.

## Data Availability

Data sharing is not applicable to this article as no new data were created or analysed in this study.
